# Machine Learning-Based Classification of Anterior Circulation Cerebral Infarction Using Computational Fluid Dynamics and CT Perfusion Metrics

**DOI:** 10.3390/brainsci15040399

**Published:** 2025-04-15

**Authors:** Xulong Yin, Yusheng Zhao, Fuping Huang, Hui Wang, Qi Fang

**Affiliations:** 1 Department of Neurology, The First Affiliated Hospital of Soochow University, 899 Pinghai Road, Suzhou 215006, China; 20237832010@stu.suda.edu.cn (X.Y.); 20245232071@stu.suda.edu.cn (Y.Z.); 20237832074@stu.suda.edu.cn (F.H.); wanghui@suda.edu.cn (H.W.); 2 Institute of Stroke Research, Soochow University, Suzhou 215006, China

**Keywords:** computational fluid dynamics, hemodynamics, intracranial atherosclerotic stenosis, machine learning, CT perfusion

## Abstract

**Background:** Intracranial atherosclerotic stenosis (ICAS) is a leading cause of ischemic stroke, particularly in the anterior circulation. Understanding the underlying stroke mechanisms is essential for guiding personalized treatment strategies. This study proposes an integrated framework that combines CT perfusion imaging, vascular anatomical features, computational fluid dynamics (CFD), and machine learning to classify stroke mechanisms based on the Chinese Ischemic Stroke Subclassification (CISS) system. **Methods:** A retrospective analysis was conducted on 118 patients with intracranial atherosclerotic stenosis. Key indicators were selected using one-way ANOVA with nested cross-validation and visualized through correlation heatmaps. Optimal thresholds were identified using decision trees. The classification performance of six machine learning models was evaluated using ROC and PR curves. **Results:** Time to Maximum (Tmax) > 4.0 s, wall shear stress ratio (WSSR), pressure ratio, and percent area stenosis were identified as the most predictive indicators. Thresholds such as Tmax > 4.0 s = 134.0 mL and WSSR = 86.51 effectively distinguished stroke subtypes. The Logistic Regression model demonstrated the best performance (AUC = 0.91, AP = 0.85), followed by Naive Bayes models. **Conclusions:** This multimodal approach effectively differentiates stroke mechanisms in anterior circulation ICAS and holds promise for supporting more precise diagnosis and personalized treatment in clinical practice.

## 1. Introduction

In cerebrovascular diseases, the incidence of ischemic stroke reaches as high as 87%, with a disability rate exceeding 50% [1,2]. Intracranial atherosclerotic stenosis (ICAS) is one of the most common causes of ischemic stroke or transient ischemic attack (TIA) worldwide [3]. One report indicates that the annual mortality rate for intracranial internal carotid artery stenosis in the anterior circulation is 12.4%, whereas the annual mortality rate for middle cerebral artery (MCA) stenosis is 6.8% [4]. Thus, investigation of the underlying mechanism of ICAS progression, prevention, and treatment will greatly decrease the incidence and mortality of ischemic stroke [5].

The classification of ischemic stroke is essential to optimize treatment. At present, the Trial of Org 10172 in Acute Stroke Treatment (TOAST) classification system is widely used, but it has limitations in mechanism-oriented stroke classification [6,7,8]. The Chinese Ischemic Stroke Subclassification (CISS) system further categorizes the potential mechanisms of ischemic stroke caused by intracranial and extracranial large-artery atherosclerosis (LAA) into four types based on modern imaging techniques: parent artery (plaque or thrombus) occlusion of penetrating artery, artery-to-artery embolism, hypoperfusion/embolus clearance impairment, and multiple mechanisms [9]. The CISS classification plays a more positive role in distinguishing stroke mechanisms in ICAS. Due to differing mechanisms, ischemic strokes caused by ICAS may require distinct treatment strategies. Additionally, the stroke mechanism is associated with different recurrence risks of stroke in patients with ICAS undergoing medical treatment [10].

In recent years, computational fluid dynamics (CFD) has been increasingly applied to simulate cerebral hemodynamics in ICAS and to investigate its clinical implications [11]. Feng et al. identified a high wall shear stress ratio (WSSR) as an independent predictor of arterial-to-arterial embolism as a stroke mechanism in patients with anterior circulation ICAS, with this association being more pronounced in patients with low pressure ratios (PRs) (large translesional pressure gradients) [12]. Additionally, Li et al. found that, among patients with medial and cortical border-zone infarctions, where hypoperfusion is commonly deemed the stroke mechanism, a low forward flow (PR ≤ median) was independently associated with medial border-zone infarction and that there was a higher incidence of small cortical infarctions in patients with cortical border-zone infarctions [13]. These studies indicate that hemodynamics play a crucial role in determining the stroke mechanism of ICAS. Studies on computed tomography perfusion (CTP) and ICAS further underscore its significance in hemodynamic analysis [14,15]. The exploration of the combination of computational fluid dynamics modeling and CT perfusion to predict the etiology of cerebral infarction is still in its infancy.

Machine learning methods have increasingly been integrated into medical research. By extracting relevant information from datasets and uncovering hidden correlations among parameters, machine learning has successfully been employed for diagnosis and prediction of ICAS-related conditions [16,17]. This study distinguishes itself from previous research that focused solely on a single imaging modality or isolated hemodynamic factors by being the first to integrate CFD parameters, CTP metrics, and anatomical features into a machine learning framework. This innovative approach enables the classification of ischemic stroke mechanisms associated with intracranial atherosclerotic stenosis in the anterior circulation based on the CISS system. The primary objective of this methodology is to improve diagnostic accuracy and to support the development of individualized treatment strategies for patients suffering from ischemic stroke.

## 2. Materials and Methods

### 2.1. Study Design and Subjects

This study included patients with ICAS stroke from October 2021 to June 2024. It was approved by the local Institutional Review Board, and informed consent was obtained from all patients. The inclusion criteria were as follows: (1) adult inpatients with atherosclerotic narrowing of the intracranial internal carotid artery or middle cerebral artery; (2) all patients who underwent diffusion-weighted MRI; (3) successful construction of a computed tomography angiography (CTA)-based CFD model; (4) all patients who underwent digital subtraction angiography (DSA) with a recorded angiographic projection angle ≥25°. Patients were excluded if they had an ischemic stroke due to non-atherosclerotic intracranial stenosis (e.g., Moyamoya disease, vasculitis, or dissection), intracranial artery ischemia, or complete occlusion, as were those with underlying cardiac conditions (e.g., atrial fibrillation) and those who had undergone angioplasty (including intracranial artery surgery, direct or indirect bypass surgery, or carotid endarterectomy) within one month prior to stroke. The baseline data collected included demographic information; admission blood pressure; Modified Rankin Scale (mRS) before onset; admission National Institutes of Health Stroke Scale (NIHSS) score; smoking history; history of hyperlipidemia, hypertension, and diabetes; ischemic heart disease; history of ischemic stroke or TIA; and blood tests, including glucose, triglycerides, Hemoglobin A1c (HbA1c), High-Density Lipoprotein (HDL), and Low-Density Lipoprotein (LDL) cholesterol.

### 2.2. Classification of Stroke Mechanisms in Patients Based on CISS

We classified the possible stroke mechanisms in symptomatic ICAS patients based on the location and severity of infarcts in Diffusion-Weighted Imaging (DWI) and the presence of ICAS lesions in MRA or CTA. According to the CISS criteria [9], stroke mechanisms can be categorized into (1) hypoperfusion, (2) artery-to-artery embolism (AAE), (3) penetrating artery occlusion due to atherosclerotic parent artery occlusion (PAO), and (4) mixed mechanisms. (To enhance diagnostic specificity, we ruled out mixed mechanisms.) Two investigators independently classified the possible stroke mechanisms, with discrepancies resolved through consultation with a third researcher. Inter-rater reliability for classification was high (kappa 0.841, 95% CI: 0.743–0.939), and intra-rater reliability was also high (kappa 0.875, 95% CI: 0.788–0.962).

### 2.3. Acquisition of CTP Data

Each patient underwent a standard multimodal CT protocol upon admission, which included non-contrast computed tomography (NCCT), single-phase CTA, and whole-brain CTP. The scans were performed using a 256-slice GE Revolution CT scanner (GE Healthcare, Milwaukee, WI, USA) with *z*-axis coverage of 16 cm. All CTP data were automatically generated using the RAPID software (v2017; iSchemaView, Menlo Park, CA, USA). Post-processing involved the use of delay- and dispersion-corrected singular value decomposition to generate the Time to Maximum (Tmax) of the Residue Function, cerebral blood flow (CBF), and cerebral blood volume (CBV).

### 2.4. CTA-Based Cerebral Hemodynamic Modeling and Quantitative Analysis

A CFD model was constructed using ANSYS software (v2022 R1) to simulate cerebral blood flow in the vicinity of symptomatic ICAS lesions and to quantify hemodynamic indices such as pressure and shear stress. The key steps were as follows: (1) reconstructing the 3D arterial geometry from CTA images, with a focus on the intracranial segments of the internal carotid artery [18], MCA, and anterior cerebral artery (ACA); (2) meshing the vascular surface and lumen, involving 0.5 to 1 million elements. The maximum size of the mesh was 0.1 mm at the inlet and outlet surfaces and 0.25 mm in the remaining parts; (3) setting boundary conditions and blood properties—the inlet pressure derived from previous pressure wire measurements, and the outlet pressure obtained from the FDA-approved AccuFFicas software (v1.0) [19]. The arterial wall was considered rigid, with a no-slip flow condition, and blood was modeled as an incompressible Newtonian fluid (viscosity: 0.0035 kg·m^−1^·s^−1^; density: 1050 kg·m^−3^); (4) solving the Navier–Stokes equations [20,21] to simulate blood flow. Detailed methodologies are documented in the prior literature.

### 2.5. Machine Learning and Modeling

The dataset was partitioned into training and testing sets with an 80:20 ratio, employing a fixed random seed (random state = 42) to ensure reproducibility. Six machine learning classifiers were utilized: Decision Tree (DT), Random Forest (RF), Support Vector Machine (SVM), K-Nearest Neighbors (KNN), Logistic Regression (LR), and Naive Bayes (NB). A soft voting classifier was employed to ensemble these models. All models were implemented using Scikit-learn with default hyperparameter settings, and feature standardization was performed via the Standard Scaler to improve the performance of models such as SVM and KNN. Performance evaluation on the test set was conducted using metrics, including accuracy, precision, specificity, recall, F1 score, and Cohen’s kappa. Comparative analysis encompassed ROC curves, PR curves, and cross-validation.

### 2.6. Statistical Analyses

For baseline data, continuous variables are presented in the form of mean ± standard deviation (mean ± SD), maximum (max), minimum (min), and interquartile range (IQR) values, while categorical variables are summarized using counts and percentages. To identify relevant characteristics for differentiating CISS subtypes and to prevent data leakage, analysis of variance (ANOVA) was performed on 80% of the training set, followed by nested cross-validation. Tukey’s HSD post hoc test was conducted on the ANOVA results to assess significant differences in hemodynamic, anatomical, and CTP parameters among different CISS groups, with the results visualized using box plots. Subsequently, the Benjamini–Hochberg procedure was applied to adjust the obtained *p*-values to control the false-discovery rate (FDR) and address issues of multiple comparisons. Features with adjusted *p*-values less than 0.05 were considered statistically significant and retained for subsequent modeling. All statistical analyses and visualizations were conducted using Python 3.13.

## 3. Results

### 3.1. Patient Characteristics

A total of 118 patients were included, including 45 (38.1%), 39 (33.1%), 21 (17.8%), and 13 (11.0%) patients with no infarction, hypoperfusion, AAE, or PAO, respectively, with mean ages of 59 years (IQR: 49.0–69.0), 59 years (IQR: 50.0–69.0), 57 years (IQR: 47.0–68.0), and 59 years (IQR: 57.0–66.0), and males accounted for 55.6%, 79.5%, 66.7%, and 69.2%, respectively. There were no significant differences in admission blood pressure, smoking, hyperlipidemia, hypertension, diabetes, ischemic heart disease, ischemic stroke or TIA, or relevant laboratory parameters, including blood glucose, triglycerides, HbA1c, HDL, and LDL-C. However, the mRS score before the onset of the disease was higher in the AAE group, the NIHSS score in the admission group was the lowest in the no infarction group, and the statistical results were significantly different; the specific results are shown in Table 1.

### 3.2. CT Perfusion, Anatomy, and Computational Fluid Dynamics Index Analysis

#### 3.2.1. Selection of Features for CISS Categorization

**Figure 1:**
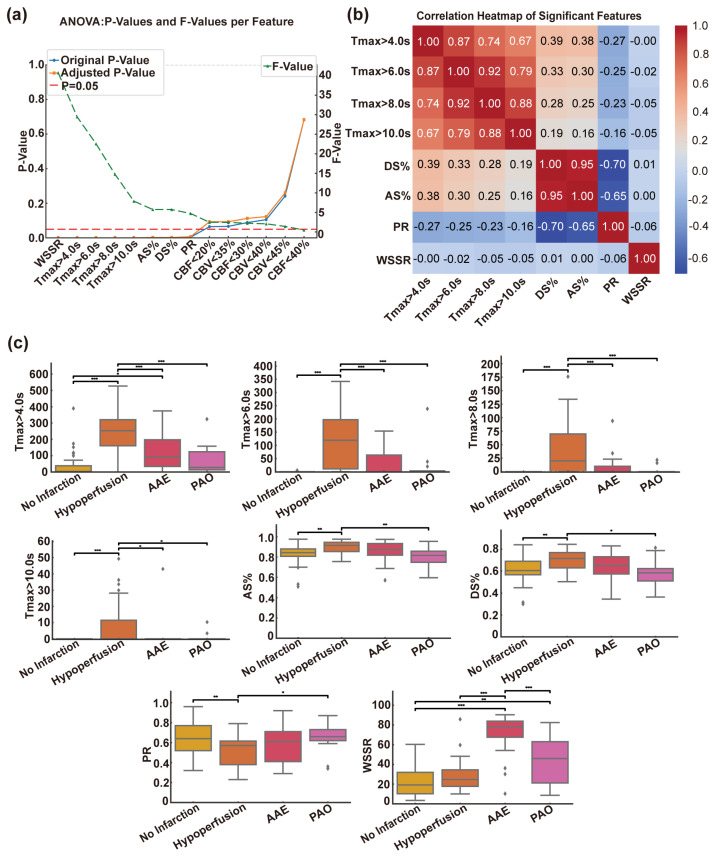
Analysis and screening of CT perfusion, anatomical, and computational fluid dynamics indices. (**a**) Results of analysis of variance (ANOVA) for all candidate features. The chart depicts the original *p*-values (blue line) and Benjamini–Hochberg-corrected *p*-values (orange line) on the left *y*-axis, along with the corresponding F-values (green dashed line) on the right *y*-axis. The red line signifies the significance threshold (corrected *p* = 0.05). (**b**) Correlation analysis results for different indicators. (**c**) One-way ANOVA box plots for different characteristics. AAE, arterial–arterial embolism; PAO, penetrating artery occlusion due to atherosclerotic parent artery occlusion; Tmax, Time to Maximum; DS%, degree of stenosis percentage; AS%, area stenosis percentage; PR, pressure ratio; WSSR, wall shear stress ratio. Statistical significance: * *p* < 0.05; ** *p* < 0.01; *** *p* < 0.001.

To identify specific imaging characteristics associated with subtypes of the CISS, we conducted a one-way ANOVA on the training set, which comprised 80% of the total dataset. A total of 14 candidate features were evaluated, including CT perfusion metrics such as Tmax > 4.0 s, Tmax > 6.0 s, Tmax > 8.0 s, Tmax > 10.0 s, CBF < 40%, CBF < 30%, CBF < 20%, CBV < 45%, CBV < 40%, and CBV < 35%, as well as anatomical metrics, including DS% and AS%, and hemodynamic metrics, including PR and WSSR. Based on the F-statistics and adjusted *p*-values from the ANOVA, we selected the top eight features. These features included WSSR, Tmax > 4.0 s, Tmax > 6.0 s, Tmax > 8.0 s, Tmax > 10.0 s, CBF < 40%, AS%, and DS%, as depicted in Figure 1a. All these features exhibited adjusted *p*-values below the 0.05 threshold and demonstrated the highest statistical discriminatory power across various CISS subgroups. To ensure the robustness of the feature selection process and to prevent data leakage, we embedded the ANOVA within a stratified nested cross-validation framework. The resulting mean cross-validation accuracy was 73.4% (±12.4%), indicating steady generalization capability and stability across folds.

#### 3.2.2. Correlation Heatmap Analysis of Various Indicators

To mitigate multicollinearity and ensure model stability, we employed a Pearson correlation heatmap to analyze pairwise correlations among the first eight selected features. As depicted in Figure 1b, strong linear correlations were observed among several perfusion parameters, particularly prominently within the Tmax series. Specifically, Tmax > 6.0 s, Tmax > 8.0 s, and Tmax > 10.0 s exhibited high correlations with Tmax > 4.0 s, suggesting potential redundancy in their representation. Similarly, a moderate correlation between DS% and AS% indicated some overlap in their contributions to the model. To prevent overfitting and multicollinearity issues, we retained only the most representative feature from each highly correlated cluster. Consequently, Tmax > 4.0 s was selected as the representative feature for the Tmax group, while AS% was preserved due to its marginally stronger statistical significance compared to DS%. The final feature set employed for model training and interpretation comprised WSSR, Tmax > 4.0 s, CBF < 40%, AS%, and PR.

#### 3.2.3. Differences in Indicators in the CISS Classification

Boxplots were utilized to visualize the distribution of eight selected features across the four CISS subtypes (Figure 1c). Regarding perfusion indices, Tmax > 4.0 s, Tmax > 6.0 s, Tmax > 8.0 s, and Tmax > 10.0 s were significantly elevated in both the hypoperfusion and AAE groups, with the overall highest values observed in the hypoperfusion group. The most pronounced intergroup differences were noted between the hypoperfusion and no infarction groups. These findings are clinically intuitive, as prolonged Tmax values reflect impaired perfusion at the tissue level. Anatomical parameters included AS% and DS%, with the hypoperfusion group exhibiting the highest rates of diameter stenosis and area stenosis, followed by the AAE group. Regarding hemodynamic indices, the hypoperfusion group had the lowest PR values, followed by the AAE group. For WSSR, values were highest in the AAE group, followed by the PAO and hypoperfusion groups, with the lowest values observed in the no infarction group. Statistically significant differences (*p* < 0.001) were noted between PAO and other groups, suggesting that elevated local shear stress may predispose to embolus detachment. These findings highlight distinct radiological feature spectra among CISS subtypes. Results for CBF < 40%, CBF < 30%, CBF < 20%, CBV < 45%, CBV < 40%, and CBV < 35% are not presented due to the lack of statistical significance. The specific comparative results can be observed in Table 2.

### 3.3. Threshold Values for Critical Indicators in CISS Typing

**Figure 2:**
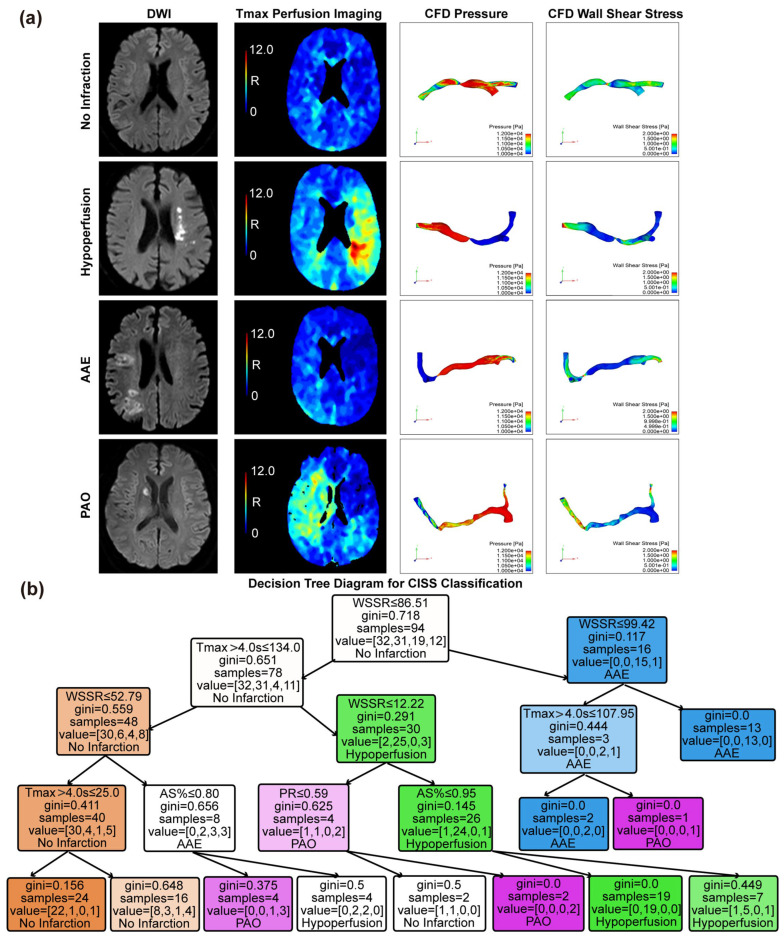
Visualization of CISS classification for cerebral infarction and threshold determination for various indicators. (**a**) Magnetic resonance DWI sequences, Tmax images, and CFD modeling-derived pressure and WSS maps are presented for the no infarction group, hypoperfusion group, AAE group, and PAO group. (**b**) Decision Tree model based on the criteria of Tmax > 4.0 s, AS%, PR, and WSSR to determine the classification thresholds for CISS. AAE, arterial–arterial embolism; PAO, penetrating artery occlusion due to atherosclerotic parent artery occlusion; Tmax, Time to Maximum; AS%, area stenosis percentage; PR, pressure ratio; WSSR, wall shear stress ratio.

Based on the CISS classification of cerebral infarction, we present the DWI sequence images, Tmax > 4.0 s perfusion maps, PR maps derived from computational fluid dynamics, and WSSR maps for representative patients of different subtypes within the anterior circulation LAA type. Specific examples can be found in Figure 2a. To determine the thresholds distinguishing various CISS subtypes, we employed a DT method, with detailed results illustrated in Figure 2b. The DT yielded critical cutoff values for each indicator: when Tmax > 4.0 s ≤ 25.0 mL and WSSR ≤ 52.80, no infarction is suggested. When Tmax > 4.0 s > 134.0 mL and 12.22 ≤ WSSR ≤ 86.51, hypoperfusion is indicated. AAE is suggested when WSSR > 86.51. When 6.95 mL < Tmax > 4.0 s ≤ 134.0 mL, 52.79 < WSSR ≤ 86.51, and AS% ≤ 0.80, the classification likely indicates a PAO.

### 3.4. Machine Learning Model Construction and Comparison

#### 3.4.1. Quantitative Assessment of Model Performance

**Figure 3:**
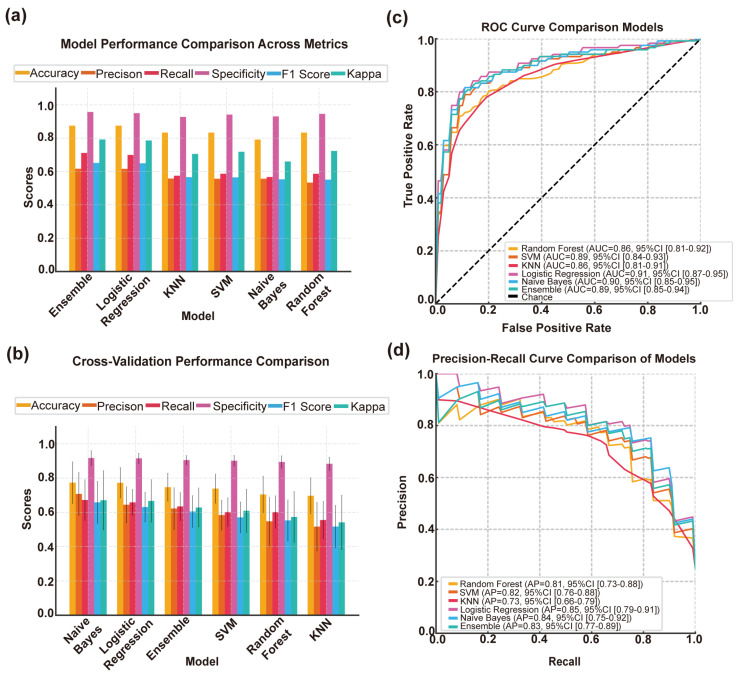
Construction and evaluation of machine learning model for CISS classification of cerebral infarction. (**a**) Comparison chart of accuracy, precision, recall, specificity, F1 score, and Kappa values across various models. (**b**) Cross-validation results of various machine learning models. (**c**) Comparison of ROC curves across various models. (**d**) Comparison of precision–recall curves across various models. SVM, Support Vector Machine; KNN, K-Nearest Neighbors; ROC, Receiver Operating Characteristic; 95% CI, 95% confidence interval.

We employed six metrics to assess model performance, including accuracy, precision, recall, specificity, F1 score, and Cohen’s kappa coefficient (Figure 3a). Within the independent test set, the ensemble model demonstrated superior performance, achieving an accuracy of 0.875, an F1 score of 0.651, and a kappa value of 0.793, exhibiting both high precision and consistency in results. The LR model showed comparable performance, with an identical accuracy of 0.875, an F1 score of 0.650, and a kappa value of 0.786, indicating strong stability and reliability. The SVM and KNN models both attained an accuracy of 0.833 but slightly lagged behind in terms of precision and F1 score. The KNN model exhibited lower consistency in distinguishing between classes (kappa value of 0.706). The NB model achieved high precision (0.557) but showed modest performance in terms of the F1 score (0.553) and kappa value (0.660), potentially constrained by the assumption of feature independence. Overall, the LR and ensemble models demonstrated the best performance and classification consistency.

#### 3.4.2. Comparison of Machine Learning Models Based on Cross-Validation Results

In the 10-fold cross-validation, we further assessed the robustness of each model across the entire dataset (Figure 3b). The NB model demonstrated the most outstanding performance, with an accuracy of 0.773 ± 0.122, an average F1 score of 0.659 ± 0.123, and a kappa value of 0.671 ± 0.173, exhibiting strong generalization capability and consistency. Following closely, the LR model achieved an accuracy of 0.773 ± 0.090, an F1 score of 0.631 ± 0.087, and a kappa value of 0.667 ± 0.124. Its performance remained stable and maintained high levels across multiple metrics. The ensemble model also showed a good balance, with an accuracy of 0.747 ± 0.082, an F1 score of 0.605 ± 0.093, and a kappa value of 0.628 ± 0.114, demonstrating the effectiveness of soft voting in combining multiple classifiers. In contrast, the KNN and RF models performed less satisfactorily across multiple metrics, exhibiting particularly significant fluctuations in performance. In summary, LR and NB models remain the preferred choices for complex multiclass classification tasks, while the ensemble model serves as a supplementary solution that balances performance and robustness.

#### 3.4.3. Comparative Analysis of Model Performance Using ROC Curves

The six models were evaluated using ROC curves and AUC values (Figure 3c). The LR model exhibited the highest AUC value (0.91, 95% CI: 0.87–0.95), demonstrating robust discriminative ability and consistency. The NB model ranked second (AUC 0.90, 95% CI: 0.85–0.95), closely followed by the SVM model (AUC 0.89, 95% CI: 0.84–0.93), both of which demonstrated stable performance. The ensemble model showed moderate effectiveness (AUC 0.89, 95% CI: 0.85–0.94), while the RF (AUC 0.86, 95% CI: 0.81–0.92) and the KNN algorithm (AUC 0.86, 95% CI: 0.81–0.91) exhibited relatively lower performance with wider confidence intervals, suggesting greater uncertainty in classification performance. Overall, the LR model remained the most reliable choice, with the NB model serving as a strong alternative option.

#### 3.4.4. Precision–Recall Analysis and Model Performance Comparison

The classification performance was further assessed using precision–recall (PR) curves and average precision (AP) values (Figure 3d). The LR model achieved the highest AP value (0.85, 95% CI: 0.79–0.91), maintaining an excellent balance between precision and recall. The NB model followed closely (AP 0.84, 95% CI: 0.75–0.92), and the ensemble model also demonstrated competitive performance (AP 0.83, 95% CI: 0.77–0.89). In contrast, the RF model (AP 0.81), SVM (AP 0.82), and the KNN algorithm (AP 0.73) had lower AP values with wider confidence intervals. Consistent with the ROC analysis results, the LR model was proven to be the most effective, while the ensemble and NB models provided stable and balanced alternative choices.

## 4. Discussion

The CISS offers more detailed insights into the pathophysiology of stroke and introduces the mechanism of ischemic stroke caused by LAA [9]. The study demonstrates that the CISS classification system is both effective and reliable, exhibiting a high degree of consistency with the TOAST classification system overall [22]. We focused on the three subtypes of LAA in the CISS classification for cerebral infarction, namely, hypoperfusion, AAE, and PAO, and analyzed the potential hemodynamic mechanisms by combining CT perfusion indices with computational fluid dynamics metrics, thereby providing guidance for the selection of treatment regimens for cerebral infarction. Patients with hypoperfusion may benefit more from interventions that improve distal blood flow. AAE patients require antiplatelet therapy aimed at stabilizing plaques to reduce the risk of embolism. For PAO patients, the emphasis is on protecting the perforating artery perfusion area to avoid further damage caused by treatment. Therefore, it is necessary to classify the etiology of cerebral infarction in patients with symptomatic intracranial atherosclerotic stenosis.

The application value of CT perfusion indices in ICAS has gradually been recognized. Yu et al. reported that patients with a volume exceeding 83 mL of tissue exhibiting a Tmax > 4.0 s may face a higher risk of recurrent stroke within one year [15]. Additionally, Tmax > 4.0 s can serve as a predictive factor for hyperperfusion syndrome (HPS) following stent implantation in ICAS patients [23]. Yan’s research also corroborates, through CT perfusion, that perioperative perfusion in ICAS surgery is associated with improvement and recurrence of stroke [24]. Among the CT perfusion indices included in our study, namely, Tmax, CBF, and CBV, the significance of Tmax as a critical diagnostic parameter was identified. Comparative analysis of CTP indices across different CISS stroke subtypes revealed that the Tmax volume in the hyperperfusion group was the highest and exhibited significant differences, indicating the most severe ischemia in the hyperperfusion group, with no significant differences observed in the CBF and CBV indices. Moreover, Tmax > 4.0 s played a pivotal role in distinguishing various CISS infarcts, which underscores the potential of Tmax > 4.0 s as a sensitive marker for hypoperfusion-related stroke mechanisms. Future research could explore the combination of CTP and CFD parameters to further enhance diagnostic accuracy.

CFD metrics play a pivotal role in the mechanistic investigation of ICAS. Our study integrates CFD with CISS classification, revealing that the PR could be used as an indicator to distinguish stroke type. The AAE group exhibited elevated WSSR, associated with shear stress-induced plaque rupture. These findings align with prior studies by Feng et al. [12] and Li et al. [13], underscoring the interplay between hemodynamics and stroke etiology. The integration of CFD modeling to quantify these parameters enhances the understanding of stroke pathophysiology and may facilitate the development of targeted therapeutic strategies.

In this study, the DT model was used to analyze the role of key cerebral perfusion indicators in the classification of lesions, and the relevant thresholds of Tmax > 4.0 s, WSSR, PR, and AS% were discussed. Among them, Tmax > 4.0 s ≤ 134.0 mL was associated with no infarction, while Tmax > 4.0 s > 134.0 was significantly associated with arterial ischemic events and partial arterial occlusion. The thresholds of WSSR (52.79 and 86.51) further distinguish the severity of lesions, and higher values may indicate an acute arterial event due to vascular instability factors such as plaque rupture. PR = 0.59 may serve as a cutoff point to distinguish no infarction from PAO. In terms of anatomical indicators, AS% = 0.80 tended to distinguish between hypoperfusion and PAO. The model performed well in the no infarction and hypoperfusion classifications (85% and 86% accuracy, respectively), but had limited ability to identify PAO. This model can quickly assist in identifying cerebral blood flow abnormalities and assessing the severity of lesions.

The machine learning models evaluated in this study demonstrated varying levels of performance in classifying stroke subtypes. The ROC and PR curve analyses provided valuable insights into the discriminative capabilities of the different models. LR emerged as the overall top performer, with an average AUC of 0.91 and an average AP of 0.85 and its narrow 95% CI indicating robust stability and generalization ability. Ensemble models, which integrate predictions from multiple base classifiers, also performed commendably, though they were marginally inferior to LR in certain metrics, benefiting from the integration of diverse algorithmic perspectives. NB also demonstrated favorable results, particularly in handling limited feature sets, despite challenges in sensitivity and class imbalance handling. In contrast, KNN and SVM showed more variable performance, potentially due to their sensitivity to feature scaling, noise, and data imbalance. These findings underscore the potential of machine learning to support accurate diagnosis in complex clinical scenarios.

Although LR demonstrated remarkable performance in this study, it is imperative to acknowledge that the input feature set derived from one-way ANOVA analysis is inherently biased towards models that assume linear relationships. These features were selected due to their significant statistical separability and may collectively contribute to their robust performance across various metrics. In contrast, other classifiers such as RF and SVM might benefit from more intricate or non-linear feature interactions, an advantage that was not specifically optimized in our current pipeline. To ensure comparability across models, we deliberately applied the same final feature set to all algorithms. However, this standardized approach may have constrained the performance potential of more complex models. Future research will explore model-specific feature selection methodologies to better harness the strengths of various classifiers in the classification of stroke mechanisms.

This study demonstrates the feasibility of integrating CFD with anatomical indices and CTP imaging parameters, coupled with machine learning techniques, for stroke mechanism classification based on the CISS system. Several areas, however, warrant further optimization. Firstly, external validation of the model utilizing larger, multicenter datasets is imperative to ascertain its generalizability across diverse populations [25,26]. Secondly, the incorporation of a broader spectrum of clinical variables, such as cardioembolic events and lacunar infarcts, could augment the robustness of the model, a point previously underscored in investigations pertaining to stroke pathophysiology [27]. Thirdly, future research should contemplate employing classifier-specific feature selection strategies rather than a uniform feature set. For instance, the Least Absolute Shrinkage and Selection Operator (LASSO) or elastic net regularization may prove beneficial for linear models, while tree-based models could leverage embedded importance metrics like SHAP values or Gini importance. Furthermore, dimensionality reduction techniques, including principal component analysis and methods based on autoencoders, may augment model performance in small-sample scenarios and elucidate latent structures within multimodal data. Finally, the integration of explainable artificial intelligence (XAI) methodologies is crucial for enhancing model interpretability and clinical acceptance, an increasingly prominent focus within stroke prediction research [28]. These strategies will contribute to refining the proposed framework and advancing the development of clinically applicable diagnostic tools tailored to specific stroke mechanisms.

## 5. Conclusions

This study validates the feasibility and efficacy of integrating CT perfusion parameters, computational fluid dynamics metrics, and machine learning algorithms for the classification of mechanisms in patients with intracranial atherosclerotic ischemic stroke. Based on the CISS classification system, key imaging and hemodynamic features, including Tmax > 4.0 s, WSSR, PR, and AS%, were identified as the most informative indicators for differentiating stroke subtypes.

Among the six machine learning algorithms evaluated, LR demonstrated the best classification performance (AUC = 0.91, AP = 0.85), followed closely by the NB model. The proposed framework provides a robust clinical decision support tool for patients with anterior circulation ICAS, enhancing diagnostic accuracy and facilitating the implementation of more personalized treatment strategies.

Future research will focus on validating this approach through larger multicenter datasets and incorporating additional clinical variables, such as comorbid cardiovascular conditions, to enhance the model’s generalization capability. Additionally, exploring feature selection and non-linear dimensionality reduction methods for specific models holds promise for optimizing the performance of complex classifiers like SVM and RF. Ultimately, this research direction aims to advance the development of intelligent, mechanism-oriented decision support systems for stroke management.

## Figures and Tables

**Table 1:** Baseline data statistics for patients.

Classification	No Infarction	Hypoperfusion	AAE	PAO	*p*-Value
	(n = 45)	(n = 39)	(n = 21)	(n = 13)	
Age	64.0 (49.0–69.0)	62.0 (50.0–69.0)	63.0 (47.0–68.0)	63.0 (57.0–66.0)	0.915
Male	25 (55.6)	31 (79.5)	14 (66.7)	9 (69.2)	0.144
SBP	138.0 (130.0–154.0)	135.0 (129.0–155.5)	135.0 (127.0–151.0)	140.0 (130.0–148.0)	0.876
DBP	79.0 (71.0–85.0)	82.0 (73.0–88.0)	80.0 (71.0–86.0)	83.0 (72.0–87.0)	0.888
mRs	2 (0.0–2.0)	2 (1.5–3.0)	3 (1.0–4.0)	1 (1.0–2.0)	<0.001
NIHSS	1 (0.0–1.0)	3 (1.0–4.0)	3 (0.0–3.0)	3 (0.0–3.0)	0.001
Relevant past medical history					
Smoking	8 (17.8)	13 (33.3)	6 (28.6)	2 (15.4)	0.326
Hyperlipidemia	21 (46.7)	23 (59.0)	12 (57.1)	7 (53.8)	0.707
Hypertension	33 (73.3)	32 (82.1)	13 (61.9)	9 (69.2)	0.392
Diabetes	16 (35.6)	14 (35.9)	5 (23.8)	7 (53.8)	0.375
Ischemic heart disease	4 (8.9)	5 (12.8)	1 (4.8)	0 (0.0)	0.477
Ischemic stroke/TIA	21 (46.7)	24 (61.5)	12 (57.1)	7 (53.8)	0.592
Laboratory test results					
Blood glucose	5.28 (4.9–5.77)	5.29 (4.68–6.59)	5.24 (4.82–5.58)	5.64 (5.12–6.5)	0.128
Triglyceride	1.22 (0.91–1.86)	1.60 (0.98–1.9)	1.44 (1.3–1.67)	1.19 (1.02–1.4)	0.571
HbA1c	6.10 (5.7–6.4)	6.30 (5.75–7.0)	5.80 (5.7–6.2)	6.30 (5.6–8.1)	0.587
HDL	1.03 (0.24)	0.95 (0.28)	0.97 (0.19)	0.95 (0.26)	0.494
LDL-C	1.88 (1.47–2.39)	1.84 (1.40–2.46)	1.79 (1.64–2.42)	1.69 (1.45–2.24)	0.831

The normality test value is expressed in terms of the mean (standard deviation), interquartile range (1st quartile–3rd quartile), or count (percentage). AAE, arterial–arterial embolism; PAO, penetrating artery occlusion due to atherosclerotic parent artery occlusion; TIA, transient ischemic attack; SBP, systolic blood pressure; DBP, diastolic blood pressure; HbA1c, glycated hemoglobin; HDL, high-density lipoprotein cholesterol; LDL-C, low-density lipoprotein cholesterol.

**Table 2:** CTP, anatomical, and hemodynamic parameters.

Classification	No Infarction	Hypoperfusion	AAE	PAO	*p*-Value
	(n = 45)	(n = 39)	(n = 21)	(n = 13)	
CT Perfusion Indices (mL)					
Tmax > 4.0 s	2.9 (0.0–35.9)	252.6 (160.5–320.4)	92 (34.0–197.3)	27.8 (13.0–123.9)	<0.001
Tmax > 6.0 s	0.0 (0.0–0.0)	119.0 (10.6–197.0)	0.0 (0.0–63.0)	0.0 (0.0–3.1)	<0.001
Tmax > 8.0 s	0.0 (0.0–0.0)	20.0 (0.0–69.9)	0.0 (0.0–10.0)	0.0 (0.0–0.0)	<0.001
Tmax > 10.0 s	0.0 (0.0–0.0)	0.0 (0.0–11.45)	0.0 (0.0–0.0)	0.0 (0.0–0.0)	<0.001
CBF < 40%	0.0 (0.0–5.4)	0.0 (0.0–19.9)	9.0 (0.0–47.4)	9.4 (0.0–37.7)	0.683
CBF < 30%	0.0 (0.0–0.0)	0.0 (0.0–0.0)	0.0 (0.0–0.004)	0.0 (0.0–0.0)	0.089
CBF < 20%	0.0 (0.0–0.0)	0.0 (0.0–0.0)	0.0 (0.0–0.0)	0.0 (0.0–0.0)	0.065
CBV < 45%	4.0 (0.0–27.6)	28.6 (0.0–48.3)	9.0 (0.0–35.7)	17.3 (0.1–31.7)	0.242
CBV < 40%	0.0 (0.0–2.5)	4.0 (0.0–8.8)	0.4 (0.0–9.0)	3.3 (0.0–10.3)	0.105
CBV < 35%	0.0 (0.0–0.0)	0.0 (0.0–0.0)	0.0 (0.0–1.6)	0.0 (0.0–2.6)	0.067
Anatomical Indicators					
DS%	0.60 (0.11)	0.70 (0.10)	0.64 (0.12)	0.58 (0.13)	0.001
AS%	0.84 (0.81–0.88)	0.91 (0.85–0.95)	0.87 (0.82–0.93)	0.82 (0.75–0.86)	0.001
Computational Fluid Dynamics Indicators					
PR	0.63 (0.16)	0.52 (0.14)	0.59 (0.18)	0.66 (0.17)	0.004
WSSR	19.2 (10.2–32.0)	24.7 (17.7–34.5)	78.3 (67.6–83.9)	45.9 (21.2–63.1)	<0.001

Normality tests are presented as means (standard deviations), interquartile ranges (first quartiles-third quartiles), or counts (percentages). AAE, artery–artery embolism; PAO, perforating artery occlusion due to atherosclerotic parent artery occlusion; Tmax, Time to Maximum; CBF, cerebral blood flow; CBV, cerebral blood volume; DS%, percentage of diameter stenosis; AS%, percentage of area stenosis; PR, pressure ratio; WSSR, wall shear stress ratio.

## Data Availability

The data are not publicly available due to privacy, but the raw data supporting the conclusions of this article will be made available by the authors on request.

## Abbreviations

ICAS	Intracranial atherosclerotic stenosis
TIA	Transient ischemic attack
MCA	Middle cerebral artery
CISS	The Chinese Ischemic Stroke Subclassification
TOAST	Trial of Organ 10172 in Acute Stroke Treatment
CFD	Computational fluid dynamics
WSSR	Wall shear stress ratio
PR	Pressure ratio
CTP	Computed tomography perfusion
CTA	Computed tomography angiography
DSA	Digital subtraction angiography
mRS	Modified Rankin Scale
NIHSS	National Institutes of Health Stroke Scale
DWI	Diffusion-Weighted Imaging
AAE	Artery-to-artery embolism
PAO	Parent artery occlusion
CBF	Cerebral blood flow
CBV	Cerebral blood volume
DT	Decision Tree
RF	Random Forest
NB	Naive Bayes
KNN	K-Nearest Neighbors
LAA	Large-artery atherosclerosis
